# Bronchopulmonary penetration of isavuconazole in lung transplant recipients

**DOI:** 10.1128/aac.00613-23

**Published:** 2023-10-03

**Authors:** Antonio F. Caballero-Bermejo, Ignacio Darnaude-Ximénez, Myriam Aguilar-Pérez, Alicia Gomez-Lopez, Aránzazu Sancho-López, Cristina López García-Gallo, Gema Díaz Nuevo, Elena Diago-Sempere, Belén Ruiz-Antorán, Cristina Avendaño-Solá, Piedad Ussetti-Gil, PBISA01‐Study Group

**Affiliations:** 1 Clinical Pharmacology Department, Hospital Universitario Puerta de Hierro-Majadahonda, Instituto de Investigación Sanitaria Puerta de Hierro-Segovia de Arana, Majadahonda, Madrid, Spain; 2 Internal Medicine Department, Mater Misericordiae University Hospital, Dublin, Ireland; 3 Respiratory Medicine Department, Lung Transplant Unit, Hospital Universitario Puerta de Hierro-Majadahonda, Instituto de Investigación Sanitaria Puerta de Hierro-Segovia de Arana, Majadahonda, Madrid, Spain; 4 Mycology Reference and Research Laboratory, National Center for Microbiology (CNM), ISCIII, Majadahonda, Madrid, Spain; Providence Portland Medical Center, Portland, Oregon, USA

**Keywords:** isavuconazole, invasive fungal infection, bronchoalveolar lavage, lung transplant, tissue penetration

## Abstract

Isavuconazole’s (ISA) pharmacokinetics was studied among lung transplant recipients to evaluate its bronchopulmonary penetration. This study included 13 patients and showed mean serum concentrations of 3.30 (standard deviation [SD] 0.45), 5.12 (SD 1.36), and 6.31 (SD 0.95) at 2 h, 4 h, and 24 h respectively. Mean concentrations in the epithelial lining fluid were 0.969 (SD 0.895), 2.141 (SD 1.265), and 2.812 (SD 0.693) at the same time points. ISA is a drug with a tolerable safety profile that achieves adequate concentrations in the lung.

## INTRODUCTION

Lung transplant recipients are especially vulnerable to the development of fungal infections as a result of the peculiarities of the pulmonary graft and the high levels of immunosuppression necessary for the prevention of rejection (1). Isavuconazole (ISA) has shown a better safety profile and non-inferiority to voriconazole in the treatment of patients with invasive fungal infection (IFI) (2, 3), which is maintained in lung transplant patients (4). There is some information about tissue penetration (e.g., brain, lungs, and ascites fluid) (5 – 7), but the available data are mostly based on animal experiments and there are no data regarding bronchopulmonary penetration of ISA.

Given that pulmonary aspergillosis is the most common presentation of IFI, it is important to understand the bronchopulmonary disposition of ISA. The aim of this study was to describe the pharmacokinetic profile of oral ISA at the bronchopulmonary level in patients receiving lung transplantation. This was a single-center, phase IV, open-label, prospective, non-controlled, single-treatment arm clinical trial. The trial was approved by the Spanish Medicines Regulatory Authority (Agencia Española de Medicamentos y Productos Sanitarios) and by the Research Ethics Committee at Hospital Universitario Puerta de Hierro-Majadahonda (registry number 167/19). Full details of the trial protocol can be found in the Supplementary Material.

Patients were eligible for enrollment if they were lung transplant recipients of at least 18 years of age and hospitalized due to clinical suspicion of IFI with medical indication for treatment with ISA and were able to meet the study procedures.

After signing the informed consent, patients were started on oral isavuconazole loading doses (200 mg/8 h for the first 48 h) followed by a maintenance dose (starting from day 3, 200 mg/24 h). During the course of treatment and after the steady state was reached, one bronchoscopy was performed per patient. Patients were randomly allocated to one of the three different intervals of time between ISA administration and bronchoscopy: 2, 4, or 24 h. Four blood samples were obtained per patient: at 72 h after treatment initiation, the day of the bronchoscopy, at the time of the bronchoalveolar lavage (BAL) (simultaneously), and at least 7 days after treatment initiation, in order to analyze ISA serum levels. ISA concentrations were measured in serum and epithelial lining fluid (ELF) by a validated high-performance liquid chromatography (HPLC)/UV coupled to fluorescence method.

The primary outcome was the measurement of ISA concentrations in ELF. ISA levels in ELF were obtained from BAL. The volume of epithelial lining fluid was estimated using the concentration of urea in serum and in BAL from the following formula (8): Estimated ELF volume = amount of total urea in BAL (mg)/serum urea concentration (mg/mL). The ratio between ELF/serum at each time point was estimated from the average values of the patients included in each group.

Secondary outcomes were the incidence and severity of adverse events evaluated. These events were categorized according to the National Cancer Institute Common Terminology Criteria for Adverse Events, version 5.0.

The final BAL volume collected per patient and time point were quantified and divided into aliquots necessary for microbiological routine study, ISA quantification, and urea determination.

Serum and BAL fluid samples were assayed for ISA quantification by a validated high-performance liquid chromatography/UV coupled to fluorescence assay technique (HPLC- photodiode array [PDA]/F; Waters Chromatography, Spain).

Between 22 October 2020 and 29 October 2021, 13 patients receiving ISA to treat suspected IFI were enrolled in the study. Baseline characteristics were evenly distributed across study arms (Table 1). All patients reported full adherence to ISA treatment during the study period. Transplantation and infection details of every patient are shown in Table 2.

**TABLE 1:** Baseline demographics by groups

Parameter	All (*n* = 13)	2 h (*n* = 3)	4 h (*n* = 5)	24 h (*n* = 2)	No bronchoscopy (*n* = 3)
Age, median (IQR), yr^*a*^	63.7 (59.1–65.5)	60.6 (59.9–63.1)	64.4 (63.7–66.3)	53.1 (49.9–56.2)	64.3 (61.8–68.2)
Male sex, no. (%)	5 (41.7)	1 (33.0)	1 (20.0)	1 (50.0)	2 (67.7)
Type of transplant					
Bilateral, n (%)	11 (84.6)	3 (100)	5 (100)	2 (100)	1 (33.3)
Unilateral, n (%)	2 (15.4)	0 (0)	0 (0)	0 (0)	2 (67.7)
Median time (IQR) from transplantation to bronchoscopy, days	224 (105–439)	385 (216.5–412)	140 (102–931)	164 (135–194)	–^*b*^
Median time (IQR) from treatment initiation to bronchoscopy, days	21 (11–28)	20 (15–24)	13 (7–28)	57 (39–74)	–

^*a*^ "IQR”, interquartile range.

^*b*^ "–”, Data not available.

**TABLE 2:** Disease characteristics^*a*^

S. No.	Age	Sex	BLTx/ULTx	Underlying disease	Type of fungal infection	Microorganism identified
1	63.7	F	BLTx	ILD	Infection of the bronchial stump	*Paecilomyces lilacinus*
2	65.5	F	BLTx	COPD	Tracheobronchitis	*Aspergillus flavus*
3	59.1	M	BLTx	COPD	Infection of the bronquial sutura	*Aspergillus fumigatus*
4	71.6	M	ULTx	ILD	Probable invasive disease	*Aspergillus fumigatus*, *Aspergillus terreus*
5	58.8	F	ULTx	ILD	Invasive disease	None
6	59.4	M	BLTx	ILD	Tracheobronchitis	*Aspergillus fumigatus*
7	64.4	M	BLTx	Pneumoconiosis	Probable invasive disease	*Aspergillus fumigatus*
8	46.7	F	BLTx	COPD	Tracheobronchitis	*Aspergillus nidulans*
9	67.2	F	BLTx	ILD	Probable invasive diseasete	*Aspergillus fumigatus*
10	64.3	M	BLTx	ILD	Invasive disease	*Acinetobacter pittii* y *Hafnia alvei*
11	66.3	F	BLTx	COPD	Probable invasive disease	*Aspergillus terreus*
12	60.6	F	BLTx	COPD	Colonization	*Circinella chinensis*
13	56.1	F	BLTx	COPD	Infection of the bronquial sutura	*Rhizopius arrhizus*

^*a*^ Bilateral lung transplantation (BLTx), unilateral lung transplantation (ULTx), Interstitial lung disease (ILD), Chronic obstructive pulmonary disease (COPD).

Ten patients completed the scheduled bronchoscopy evaluation and were randomly assigned at different times for the realization of BAL, resulting in three patients at 2 h after the last dose of ISA, five patients at 4 h after the last dose, and two patients at 24 h after the last dose of ISA. All patients received loading doses of 200 mg isavuconazole every 8 h for the first 48 h and at least six doses of isavuconazole 200 mg/24 h prior to BAL.

**Fig 1:**
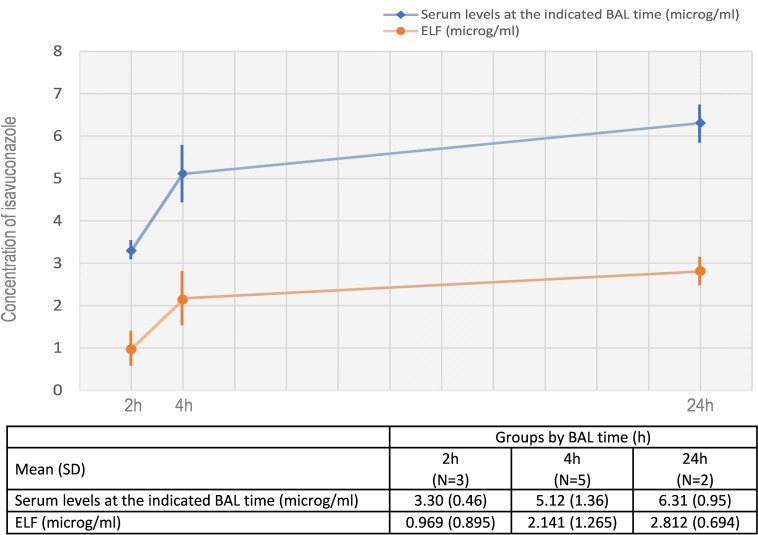
Concentrations of ISA in serum and ELF at the time of BAL (mean and IQR concentrations).

The mean trough serum concentration of ISA was 3.61 (standard deviation [SD] 1.73), 4.06 (SD 1.89) and 4.19 (2.12) on days 4 and 7 of treatment and the bronchoscopy day, respectively. The concentrations of ISA in serum, ELF, and the ELF/serum ratios at the time of BAL are summarized in Fig. 1. At the time of bronchoscopy, all patients had plasma levels of isavuconazole > 2 mg/L. The mean ELF/serum ratio was 0.283 (SD 0.242), 0.489 (standard deviation [SD] 0.372), and 0.442 (DS 0.044) at 2 h, 4 h, and 24 h, respectively.

Isavuconazole was well tolerated. The study drug had no clinically relevant effect on blood chemistry, hematology, urinalysis, or vital signs.

In this report, we assessed the bronchopulmonary penetration of oral ISA (with an oral bioavailability of 98%) at a steady state and demonstrate that ISA adequately penetrates the ELF.

The steady-state serum pharmacokinetics for ISA in this study was similar to those published previously in studies conducted in healthy volunteers (9). There are no published studies examining its bronchopulmonary penetration besides radiolabeled isavuconazonium in rats (6). In one study looking at bronchopulmonary penetration of isavuconazole into ascitic fluid (5), it was observed that ISA concentrations in ascitic fluid were lower than those seen in serum, which correlates with the findings in our study that shows lower ISA concentrations in ELF than in serum.

Given that ISA is one of the treatment options for invasive aspergillosis (2), an important consideration for each option is how well the antifungal agent penetrates the target infection site and its disposition once it is there. Pulmonary disposition of other triazoles has been reported in other studies with voriconazole (10, 11) and posaconazole (12, 13), but there are no similar studies performed with isavuconazole (14). We have to consider that there are other physiological factors, such as the presence of inflammation and changes in vascularization, which might significantly influence the exposure of drugs at the site of action (15). This aspect is especially relevant in lung transplant recipients in which the absence of bronchial revascularization and local inflammatory phenomena secondary to ischemia and reperfusion can hinder tissue penetration of drugs (1). We have to take into account that once invasive aspergillosis is well established as a pneumonic process, distribution into the ELF compartments may not necessarily be predictive of response as a drug may be present at a site but at a concentration beneath the threshold required for activity, located in the wrong subcompartment, or not biologically available. However, the overall success of treatment with ISA in cases of IFI depends on the interplay of susceptibility of the isolated species, the immune status of the host, and possibly other inter-related factors, which have to be investigated in further clinical studies.

To the best of our knowledge, this study is the first well-designed study to analyze the bronchopulmonary penetration of ISA in steady state in lung transplant recipients.

Our study has some limitations. Firstly, empirical treatment with isavuconazole was initiated based on suspicion of IFI. Secondly, despite the planned sample size being 12 patients, an additional patient was included prior to closing recruitment, resulting in a final sample size of 13. Out of the entire sample, bronchoscopy was conducted in only 10 patients, as it was not deemed clinically necessary in three of them. Moreover, despite the low sample size, the results are consistent with the initial hypothesis and in line with preliminary studies conducted on animal models. Given the characteristics of the drug, large variations in the results are not expected. Furthermore, this preliminary study was conducted with a small sample size due to the difficulty in recruiting patients (i.e., the patients could not derive any clinical benefit from their participation).

In conclusion, ISA adequately penetrated the ELF, with a relative concentration lower than that of blood. It is a drug with a tolerable safety profile that achieves adequate concentrations in the lung. These data support the use of ISA for the treatment of invasive aspergillosis, as well as the development of further studies to advance the knowledge of its therapeutic properties.

## SUPPLEMENTAL MATERIAL

Text S1 aac.00613-23-s0001.pdf Protocol.

Table S1 aac.00613-23-s0002.docx Concentrations for each patient.

ASM does not own the copyrights to Supplemental Material that may be linked to, or accessed through, an article. The authors have granted ASM a non-exclusive, world-wide license to publish the Supplemental Material files. Please contact the corresponding author directly for reuse.
