# Supplementary material for: Bronchopulmonary penetration of isavuconazole in lung transplant recipients
Source: Antimicrob Agents Chemother. 2023 Oct 3;67(10):e00613-23. doi: 10.1128/aac.00613-23 (PMC10583689; doi:10.1128/aac.00613-23)
Supplement: Text S1 — Protocol. [file aac.00613-23-s0001.pdf]

PHASE IV, UNICENTRIC, MULTIPLE DOSE, CLINICAL TRIAL, WITH A SINGLE TREATMENT ARM TO EVALUATE THE BRONCHOPULMONARY PENETRATION OF ISAVUCONAZOL IN PULMONARY TRANSPLANT RECIPIENTS

STUDY PROTOCOL

Study code: PB-ISA-01
EudraCT Number: 2019-004240-30
Version: V1.0
Date 10/31/2019

C O N F I D E N T I A L

Sponsor:
Dra. Belén Ruiz Antorán
Clinical Pharmacology Department
Hospital Universitario Puerta de Hierro Majadahonda
c/ Manuel de Falla 1, 28222-Majadahonda, Madrid
Tel: +34.91.1917479, Fax: +34.91.1917650
Email: mariabelen.ruiz@salud.madrid.org

Sponsor's representative:
Dr. Antonio Francisco Caballero Bermejo
Clinical Pharmacology Department
Hospital Universitario Puerta de Hierro Majadahonda
c/ Manuel de Falla 1, 28222-Majadahonda, Madrid
Email: afcaballerobermejo@gmail.com

Principal Investigators:
Dra. Piedad Ussetti Gil.
Pneumology Department.
Hospital Universitario Puerta de Hierro Majadahonda
c/ Manuel de Falla 1, 28222-Majadahonda, Madrid
Tel: +34.91.1916481, Fax: +34.91.1917650
Email: mariapiedad.ussetti@salud.madrid.org

Dra. Belén Ruiz Antorán
Clinical Pharmacology Department
Hospital Universitario Puerta de Hierro Majadahonda
c/ Manuel de Falla 1, 28222-Majadahonda, Madrid
Tel: +34.91.1917479, Fax: +34.91.1917650
Email: mariabelen.ruiz@salud.madrid.org

The information and data included in this protocol is the property of the Sponsor. No person is authorized to make such information public without the written permission of the Sponsor. These restrictions will also apply to all information provided in the future and considered privileged or confidential. This material may be disclosed and used by your team and associates, to the extent that it may be necessary for the development of the clinical study.

SIGNATURE PAGE

Sponsor

Belén Ruiz Antorán MD, PhD.

Clinical Pharmacology Department, Hospital Universitario Puerta de Hierro Majadahonda

C/ Manuel de Falla 1, 28222-Majadahonda, Madrid

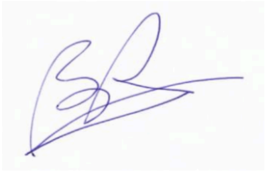

____

(Signature) (10/31/2019)

Principal Investigator

Piedad Usseti Gil, MD, PhD

Pneumology Department, Hospital Universitario Puerta de Hierro Majadahonda

c/ Manuel de Falla 1, 28222-Majadahonda, Madrid

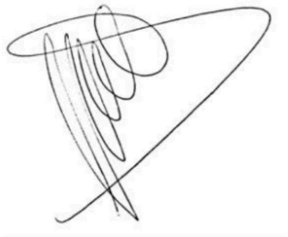

____

(Signature) (10/31/2019)

Belén Ruiz Antorán MD, PhD.

Clinical Pharmacology Department, Hospital Universitario Puerta de Hierro Majadahonda

C/ Manuel de Falla 1, 28222-Majadahonda, Madrid

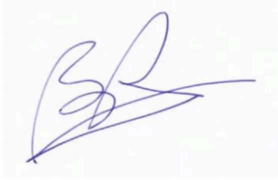

____

(Signature) (10/31/2019)

We agree to conduct this Study in accordance with the requirements of this document (the Clinical Study Protocol), the Study Reference Manual and in accordance with Good Clinical Practice and European Union Directives No 536/2014 about Clinical Trials (April, 16th 2014)

CONTACT INFORMATION

Sponsor	**Dra. Piedad Usseti Gil.**<br>Pneumology Department.<br>Hospital Universitario Puerta de Hierro Majadahonda<br>c/ Manuel de Falla 1, 28222-Majadahonda, Madrid<br>e-mail: mariapiedad.ussetti@salud.madrid.org
Sponsor's representative:	**Dr. Antonio Francisco Caballero Bermejo**<br>Clinical Pharmacology Department<br>Hospital Universitario Puerta de Hierro Majadahonda<br>c/ Manuel de Falla 1, 28222-Majadahonda, Madrid<br>e-mail: afcaballerobermejo@gmail.com
Monitoring	**Unidad de Investigación Farmacológica del Instituto de Investigación Sanitaria Puerta de Hierro-Segovia de Arana**<br>C/ Manuel de Falla,1<br>28220, Majadahonda, Madrid, España<br>e-mail: farmacologia_clinica@idiphim.org
Study Statistician	**Dra. Belén Ruiz Antorán**<br>Hospital Universitario Puerta de Hierro<br>Clinical Trial Unit; Clinical Pharmacology Department<br>C/ Manuel de Falla,1<br>28220, Majadahonda, Madrid, España<br>Tel.:911 91 64 81<br>e-mail: mariabelen.ruiz@salud.madrid.org
Laboratory	Department of Biochemistry<br>Hospital Universitario Puerta de Hierro<br>C/ Manuel de Falla,1<br>28220, Majadahonda, Madrid, España<br>Tel.: 911 91 64 81<br><br>Laboratorio de Referencia e Investigación en Micología<br>Centro Nacional de Microbiología (CNM); Instituto de Salud Carlos III<br>Carretera de Majadahonda a Pozuelo, Km 2.<br>CP 28220 - Majadahonda (Madrid, España).<br>Tel.: 918 22 34 26, 8223711

ABBREVIATIONS

ACHC Average Corpuscular Hemoglobin Concentration
ACV Average corpuscular volume
AE Adverse event
ALT Alanine transaminase
APV Average platelet volumen
AST Aspartate aminotransferase
AT axillary temperature
BAL Bronchoalveolar lavage
BP Blood pressure
BQ Biochemistry
CA Alveolar macrophages
CEIm Clinical Research Ethics Committee with Medicines
CG Coagulation
ECG Electrocardiogram
ELF Epithelial Lining Fluid
EMA European Medicines Agency
GCP Good Clinical Practice
GGT Gamma glutamyl transferase
HG Hemogram
HR heart rate
IC Informed Consent
IFD Infection / Invasive fungal disease
IHC International Harmonization Conference
LDH Lactate Dehydrogenase
MCH Mean corpuscular hemoglobin
MIC Minimum inhibitory concentration
MNI Medication not under investigation
PCRF Product in clinical research phase
PHI Protected Health Information
PI Product under investigation
PV Pharmacovigilance
RP-HPLC Reverse phase high resolution liquid chromatography
RT Respiratory Rate
SAE Serious Adverse Event
SAR Serious Adverse reaction
SERMAS Servicio Madrileño de Salud
SOT Solid Organ Transplant
SUSAR Serious and unexpected adverse reaction
UAR Unexpected adverse reaction

SUMMARY OF THE CLINICAL TRIAL

**Title of the study**	**PHASE IV, UNICENTRIC, MULTIPLE DOSE, CLINICAL TRIAL, WITH A SINGLE TREATMENT ARM TO EVALUATE THE BRONCHOPULMONARY PENETRATION OF ISAVUCONAZOL IN PULMONARY TRANSPLANT RECIPIENTS**
**EudraCT. no.**	2019-004240-30
**Type of study**	Phase IV
**Design**	Single center, phase IV, prospective, non-controlled, open-label, single treatment arm (isavuconazole) aimed at assessing/describing the pharmacokinetic profile of oral isavuconazole at the bronchopulmonary level. This will be done through the degree of penetration in the epithelial lining fluid and the alveolar macrophages from lung transplant recipients diagnosed with invasive fungal disease.<br>A total of 12 patients over 18 years of age, lung transplant recipients with IFD diagnosis and indication for isavuconazole treatment, according to the Summary of Product Characteristics.<br>Participants will start treatment with isavuconazole orally at the doses and dosage regimen listed in the Summary of Product Characteristics.<br>After 4 days of treatment with isavuconazole, patients will be randomized to perform a scheduled bronchoscopy at different times (2, 4, 8 or 12 hours) from the administration of Isavuconazole.
**Participants**	A total of 12 patients, over 18 years of age, lung transplant recipients with IFD diagnosis and indication for isavuconazole treatment according to the Summary of Product Characteristics will be included.
**Clinical sites**	One: Hospital Universitario Puerta de Hierro Majadahonda
**Follow-up**	2 weeks after inclusion
**Study duration**	A total study duration of 18 months is expected
**Main Objetive**	Describe the pharmacokinetic profile of isavuconazole at the bronchopulmonary level through the degree of penetration into the epithelial lining fluid and the alveolar macrophages in patients receiving lung transplants diagnosed with invasive fungal disease.
**Secondary Objectives**	Establish the relationship between pulmonary and systemic isavuconazole exposure.<br>To evaluate the potential pharmacokinetic interaction of isavuconazole with immunosuppressive drugs (tacrolimus and mycophenolate). This interaction will be studied in the steady state of the drugs evaluated.<br><br>Evaluate the safety and tolerability of isovuconazole.
**Primary Endpoint**	The concentrations of isavuconazole in plasma, epithelial lining fluid (ELF) and in alveolar macrophages (CA) will be determined by reverse phase HPLC (RP-HPLC).<br>The AUC of isavuconazole will be estimated at the ELF level and in CAs. Through the use of non-compartmental analyzes, the concentration-time-dependent curves in ELF and CA will be calculated.<br>The ratio between ELF / plasma and CA / plasma will be estimated from the average of the values of the patients included in each group.<br>Likewise, the percentage of the dose range with CA concentrations above the MIC 90 of isavuconazole will be calculated.
**Secondary Endpoints**	Interaction Evaluation<br>Tacrolimus and mycophenolate concentrations will be determined before and after 72 hours, 96 and 7 days after the start of treatment with isavuconazole.<br>The percentage of patients who required dose adjustment of the immunosuppressive drug after the start of treatment with isavuconazole will be determined.

	<u>Systemic Safety Assessment</u><br>It will be evaluated according to the registry of adverse effects, the physical examination of the patients, and the result of the analytical studies.
**Inclusion Criteria**	1. Lung transplant recipients over 18 years, with indication of treatment with isavuconazole.<br>2. Will and ability to meet scheduled visits, plan of treatment, laboratory analysis and other study procedures.<br>3. They must be legally competent and able to understand, sign and date the informed consent form.<br>4. Signature of the written informed consent in accordance with IHC / GCP and local legislation, obtained before any study procedure.
**Exclusion criteria**	1. Allergy or intolerance to isavuconazole.<br>2. Contraindication for bronchoscopy and / or BAL.<br>3. Any clinical condition, and / or analytical alteration that, in the opinion of the Investigator, be considered clinically significant as to participate in the study.<br>4. Individuals who show inability to follow the instructions or collaborate during the development of the study.<br>5. Women with positive pregnancy test resulto or during breastfeeding period<br>6. Have participated in another clinical trial during the 3 months prior to beginning of the study in which a research drug or a drug was tested commercially available medication.<br>7. Lack of intent or inability to follow procedures described in the protocol.<br>8. Inability to grant written informed consent.
**Study medicinal product**	CRESEMBA, 100 mg capsules (Isavuconazole)<br><br>Each capsule contains 100 mg of isavuconazole (such as 186.3 mg of isavuconazonium sulfate)
**Pharmaceutical form**	Capsules
**Dose**	Isavuconazole will be administered orally at the doses and dosage regimen<br><br>contained in the summary of product characteristics<br><br>- Loading dose: 200 mg / 8h the first 48 hours.<br><br>-Maintenance dose from day 3: 200 mg / 24h
**Administration route**	Route of administration: Oral administration.
**Control**	Not applicable
**Statistical analysis**	Descriptive

TABLE OF CONTENTS

SIGNATURE PAGE 2
CONTACT INFORMATION 3
ABBREVIATIONS 4
SUMMARY OF THE CLINICAL TRIAL 5
1 BACKGROUND AND JUSTIFICATION OF THE STUDY 11
2 OBJECTIVES 13
2.1 Main objective 13
2.2 Secondary objectives 13
3 STUDY OUTCOME MEASURES 13
3.1 Primary endpoint 13
3.2 Secondary variables 14
4 STUDY DESIGN 15
4.1 Type of Study 15
4.1.1 *Expected duration* 16
4.2 Definition of end of study 16
4.3 Criteria for premature completion of the study 16
5 PATIENTS SELECTION 17
5.1 Study population 17
5.2 Selection criteria 17
5.2.1 Inclusion criteria 17
5.2.2 Exclusion criteria 18
5.3 Selection failures 18
5.4 Drop-out or withdrawal of the subject 18
5.4.1 Criteria for definitive discontinuation of the drug under study 18
5.4.2 Withdrawal from the study 18
6 TREATMENT 19
6.1 Assignment of subjects to treatments and masking 19
6.1.1 Treatment groups 19
6.1.2 Treatment assignment method 19
6.1.3 Masking 19
6.2 Research treatment 19
6.2.1 Presentation of the product under investigation 19
6.2.2 Dose regimen 20

6.2.3 Duration of treatment ... 20
6.2.4 Treatment administration mode ... 20
6.2.5 Preparation and dispensing of study medication. ... 20
6.2.6 Labeling and packaging of study medication ... 20
6.2.7 Supply and storage of medication ... 21
6.3 Prior and concomitant treatments ... 21
6.3.1 Prior medication ... 21
6.3.2 Concomitant treatment allowed ... 21
6.3.3 Prohibited concomitant therapy ... 21
6.4 Treatment accountability and compliance with study medication ... 21
6.4.1 Method for the evaluation of therapeutic compliance ... 21
7 STUDY PROCEDURES ... 21
7.1 Calendar of visits and study flow ... 21
7.2 Selection (days –1 to 0 days) ... 23
7.2.1 Summary of the selection period ... 23
7.2.2 Selection procedures ... 23
7.3 Induction period and treatment maintenance period (day 1 to 14) ... 23
7.3.1 Summary of the treatment period ... 23
7.3.2 Procedures during treatment visits ... 23
8 PHARMACOVIGILANCE ... 26
8.1 Collection and notification of adverse events ... 26
8.2 Adverse event ... 26
8.2.1 Definitions ... 26
8.2.2 Intensity evaluation ... 27
8.2.3 8.2.3 Causality assessment ... 27
8.2.4 Measure adopted in relation to the drug under study ... 28
8.2.5 Other measures taken for the event ... 28
8.2.6 Outcome of the adverse event ... 28
8.3 Safety notifications - Procedure for Investigators ... 29
8.4 Procedures for the immediate notification of serious and unexpected adverse reactions by the Sponsor ... 30
8.5 Analytical tests ... 30
8.6 Vital signs ... 31
8.7 Electrocardiograms ... 31
8.8 Physical exams ... 31
8.9 Other explorations ... 31

9 DATA COLLECTION AND MANAGEMENT 31
9.1 Confidentiality of data 31
9.2 Center monitoring............ 31
9.3 Data collection 32
9.4 Database management and quality control............ 32
10 STATISTICAL METHODS AND DATA ANALYSIS............ 33
10.1 Analysis groups 33
10.2 Study population data: demographic characteristics and other selection characteristics............ 33
10.3 Treatments (study treatment, concomitant therapies, compliance)............ 33
10.4 Main objective 33
10.4.1 Variable 33
10.4.2 1Statistical hypothesis, model and method of analysis 33
10.4.3 Handling of unavailable values / withdrawal 34
10.5 Secondary objectives 34
10.5.1 Interaction evaluation............ 34
10.5.2 Analysis of Adverse Events............ 34
10.5.3 Analysis of analytical tests 34
10.5.4 Analysis of vital signs............ 34
10.5.5 Electrocardiogram analysis 35
10.5.6 Analysis of physical examinations............ 35
10.6 Calculation of sample size............ 35
10.7 Statistical analysis process............ 35
11 ETHICAL CONSIDERATIONS AND ADMINISTRATIVE PROCEDURES 35
11.1 Ethical and regulatory compliance............ 35
11.2 Responsibilities of the Investigator............ 35
11.3 Informed consent procedures 36
11.4 Study termination 37
11.5 Publications of the study protocol and the results 37
11.6 Dissemination of data or results 37
11.7 Documentation of the study, conservation and management of documents related to the study 37
11.8 Confidentiality of study documents and participant records 38
11.9 Inspections and audits 38
12 FUNDING AND INSURANCE............ 39
12.1 Civil Liability Insurance............ 39

12.2 Economic aspects 39
12.2.1 Economic aspects 39
12.2.2 Economic compensation to participants........ 39
13 ADHESION TO THE PROTOCOL........ 39
13.1 Modifications to the protocol 39
14 REFERENCES 40

1 BACKGROUND AND JUSTIFICATION OF THE STUDY

Invasive fungal infections (IFDs) are a frequent cause of morbidity and mortality in solid organ transplant recipients (SOT). Lung transplant recipients areespecially vulnerable to the development of these infections as a result of the peculiarities of the pulmonary graft, permanent contact with the external environment and the high levels of immunosuppression necessary for the prevention of rejection (1).
Aspergillosis is the most frequently observed IFD in tlung transplant recipients (2). The infection occurs after inhalation of spores that proliferate freely in the presence of bronchial ischemia and immunosuppression. Despite the universal prophylaxis with voriconazole and / or nebulized amphotericin B, a non-negligible percentage of patients present with Aspergillus infection (3-4). The period of greatest risk is the immediate post-transplant. However, the risk is maintained throughout the post-transplant evolution, and especially during the episodes of increased immunosuppression as a result of acute and chronic rejection treatment.
Triazoles are effective drugs against Aspergillus. Voriconazole is the drug of first choice for the treatment of invasive Aspergillosis (5-6). However, it is not free of adverse effects at liver, eye, neurological and skin levels. Its metabolism through cytochrome P450 3A4 / 5 and 2C19 determines marked interference with immunosuppressive drugs that use the same metabolic pathway, especially tacrolimus and cyclosporine.
Isavuconazole has shown a better safety profile and not inferiority to voriconazole in the treatment of patients with IFD (7,8). Isavuconazole, like the rest of triazoles, acts as a substrate and inhibitor of cytochrome CYP3A4, which implies that its administration together with other CYP3A4 substrates can cause interference with their metabolism. In this sense, studies in healthy volunteers have shown that after the administration of isavuconazole there was an increase in exposure (measured as AUC0) of tacrolimus, sirolimus, and cyclosporine of 125%, 84% and 29%, respectively. In the case of mycophenolic acid and prednisolone, the increase was of 35% and 8%. The Cmax of tacrolimus, sirolimus, and cyclosporine increased with co-administration of isavuconazole (by 42%, 65% and 6%, respectively), while that of mycophenolic acid and prednisolone decreased 11% and 4%, respectively. However, the impact of isavuconazole on the pharmacokinetics of these immunosuppressants was moderate and less than that observed with the rest of triazoles (9,10).
The relationship between plasma levels (C) and dose (D) of tacrolimus before and after isavuconazole discontinuation has been studied in 55 organ transplant recipients (20 renal, 18 hepatic, 9 cardiac and 8 pulmonary). The median C / D of tacrolimus was higher during treatment with isavuconazole and the average dose of tacrolimus had to be increased 1.3 times after drug discontinuation to maintain similar levels of immunosuppression (11).
In vitro studies have shown that the efficacy of isavuconazole depends on the fungal exposure to the drug and the MIC of the microorganism. In order to achieve the desired therapeutic effect in lung infections, it is necessary to ensure that the drug penetrates properly at the bronchopulmonary level. As it has already been shown for other azoles compounds/products,

the physicochemical properties of isavuconazole will greatly determine its distribution to tissues, organs, compartments and fluids (12).

The pharmacokinetics of isavuconazole has been studied as an experimental treatment of invasive aspergillosis in neutropenic rats. In one study (13), those subjects treated with isavuconazole had a lower residual fungal load after treatment, lower lung damage, greater survival and lower levels of (1-> 3) B-D-glucan both in serum and in BAL.

Other physiological factors such as the presence of inflammation and changes in vascularization significantly influence the exposure of drugs at the site of action (14). This aspect is especially relevant in the lung transplant recipient in which the absence of bronchial revascularization and local inflammatory phenomena secondary to ischemia and reperfusion can hinder tissue penetration of drugs.

The main disorders that lead to lung transplantation are chronic obstructive pulmonary disease (COPD), interstitial lung diseases (PID), cystic fibrosis (CF), alpha 1 antitrypsin deficiency and idiopathic pulmonary hypertension. CF patients have many peculiarities at the pharmacokinetic level. The pharmacokinetics of the azoles is altered in those patients with CF who have undergone lung transplantation, so it is often necessary to increase the dose to reach therapeutic concentrations. In the opinion of the investigators of the present study, there is insufficient evidence in published literature at present about the isavuconazole pharmacokinetics at the bronchopulmonary level in lung transplant recipients with cystic fibrosis. There are no clinical studies that analyze the bronchopulmonary penetration of isavuconazole. In this sense, bronchoalveolar lavage (BAL) performed routinely in the follow-up of lung transplant recipients, constitutes an opportunity window to analyze the bronchopulmonary penetration of isavuconazole. The simultaneous determination of the levels of isavuconazole in plasma and in the epithelial lining fluid and the alveolar macrophages obtained through the BAL, can be useful to determine its penetration at the bronchopulmonary level and correlate it with the response to treatment (16). This aspect is especially relevant as the lung transplant recipient is a population especially susceptible to the development of IFI.

The proposal of this study is to prospectively analyze the bronchopulmonary penetration of isavuconazole in a steady-state situation in patients receiving a lung transplant and analyze the interactions of said drug with immunosuppressants usually used to prevent rejection in these patients.

Hypothesis

The bronchopulmonary penetration of Isavuconazole in lung transplant recipients with suspected IFD may be compromised as a result of poor vascularization and local inflammatory phenomena. The bronchopulmonary drug penetration rate has a potential prognostic value of the response to treatment.

2 OBJECTIVES

2.1 Main objective

The main objective of this study is to describe the pharmacokinetic profile of oral isavuconazole at the bronchopulmonary level through the degree of penetration in the epithelial lining fluid and the alveolar macrophages in patients receiving lung transplantation with a diagnosis of invasive fungal disease.

2.2 Secondary objectives

- To establish the relationship between the pulmonary and systemic exposure of Isavuconazole.
- To evaluate the potential pharmacokinetic interaction of Isavuconazole with immunosuppressive drugs (tacrolimus and mycophenolate). This interaction will be studied in the steady state of the drugs evaluated.
- To evaluate the safety and tolerability of Isavuconazole.

3 STUDY OUTCOME MEASURES

3.1 Primary endpoint

<u>Isovuconazole Lung Penetration</u>

Isavuconazole concentrations will be measured in plasma, epithelial lining fluid (ELF) and alveolar macrophages (CA) by reverse phase HPLC (RP-HPLC).

- Serum isavuconazole levels. Four samples will be obtained per patient: at 72 h after treatment initiation, the day of the bronchoscopy, at the time of the BAL (simultaneously) and 7 days after treatment initiation.
- Isavuconazole levels in epithelial lining fluid (ELF) and alveolar macrophages (CA), will be obtained from bronchoalveolar lavage (BAL).

Data from the cell count, percentage of alveolar cells and volume of cell lining fluid will be presented for the different times when bronchoscopy is performed.

The volume of cell lining fluid will be estimated using the concentration of urea in plasma and in BAL from the following formula:

Estimated ELF volume = Amount of total UREA in BAL (mg) / plasma UREA concentration (mg / mL)

The AUC of isavuconazole will be estimated at the ELF level and in CAs. The concentration-time-dependent curves in ELF and CA will be calculated using non-compartmental analyzes.

The ratio between ELF / plasma and CA / plasma will be estimated from the average values of the patients included in each group.

Pharmacodynamics

The percentage of time of the drug administration interval in which the concentrations of isavuconazole in ELF and CAs in the different lung secretions are above the minimum inhibitory concentration (MIC90) will be estimated.

3.2 Secondary variables

Interaction Evaluation

Tacrolimus and mycophenolate concentrations will be determined before treatment and at 72 hours, 96 hours and 7 days from the initiation of isavuconazole treatment.

The percentage of patients who required dose adjustment of the immunosuppressive drug after the start of treatment with isavuconazole will be determined.

Assessment of the Systemic Safety

In accordance with EMA's recommendations (EMA / CPMP / EWP / 280/96 Corr1), systemic safety will be assessed by recording adverse effects, physical examination of patients, and analytical results.

- Adverse events

A systematic description of all adverse events recorded during the follow-up will be performed. Listings of adverse events, previously coded by the MedDRA dictionary, grouped by organ/systems, according to severity, intensity and causal relationship with the study drugs will be included.

In each of the planned interviews, patients will be questioned about the occurrence of any adverse events since their previous visit and the course of adverse events reported in prior visits.

All adverse events, as well as the actions taken and the results of the follow-up, will be recorded in the corresponding pages of the CRF. Such recording should be done concisely, using conventional medical terms. Recorded adverse events should not be testing, clinical assessments or determinations, eg, laboratory results or vital signs, but should reflect the diagnosis derived from the observed alteration.

- Physical examination

A systematic description of all physical examination findings will be done at the following times: start of study treatment, 7 days and 14 days

- Electrocardiogram

A systematic description of all the changes (from the screening visit) and electrocardiographic changes at the final visit (14 days after the first administration) will be performed.

- Lab tests

A systematic description of all analytical findings observed at the final visit of the study (14 days after the first administration) will be performed.

The following lab test variables will be collected:

- Complete blood count (erythrocytes, hemoglobin, hematocrit, MCV, MCH, MCHC, platelets, MPV, leukocytes, neutrophils, eosinophils, basophils, lymphocytes, monocytes);
- Coagulation tests (prothrombin time, APTT, fibrinogen); and
- General biochemistry tests, includingliver profile (Na +, K +, Cl-, Magnesium, glucose, urea, creatinine, Albumine, total bilirubin, AST, ALT, GGT, LDH and alkaline phosphatase).

4 STUDY DESIGN

4.1 Type of Study

This is a single center, phase IV, prospective, non-controlled, open-label, single treatment arm (isavuconazole) aimed at assessing/describing the pharmacokinetic profile of oral isavuconazole at the bronchopulmonary level. This will be done through the degree of penetration in the epithelial lining fluid and the alveolar macrophages from lung transplant recipients diagnosed with invasive fungal disease.

A total of 12 patients over 18 years of age, lung transplant recipients with IFD diagnosis and indication for isavuconazole treatment,according to the Summary of Product Characteristics.

Participants will start treatment with isavuconazole orally at the doses and dosage regimen listed in the Summary of Product Characteristics:

- Loading dosis: 200 mg / 8h, the first 48 hours.
- Maintenance dose from day 3: 200 mg / 24h.

After 4 days of treatment with isavuconazole, patients will be randomized to perform a scheduled bronchoscopy at different times with the following distribution:

Group 1, n = 3 patients: time 2 hours from the administration of Isavuconazole.
Group 2, n = 3 patients: time 4 hours from the administration of Isavuconazole.
Group 3, n = 3 patients: time 8 hours from the administration of Isavuconazole.
Group 4, n = 3 patients: time 12 hours from the administration of Isavuconazole

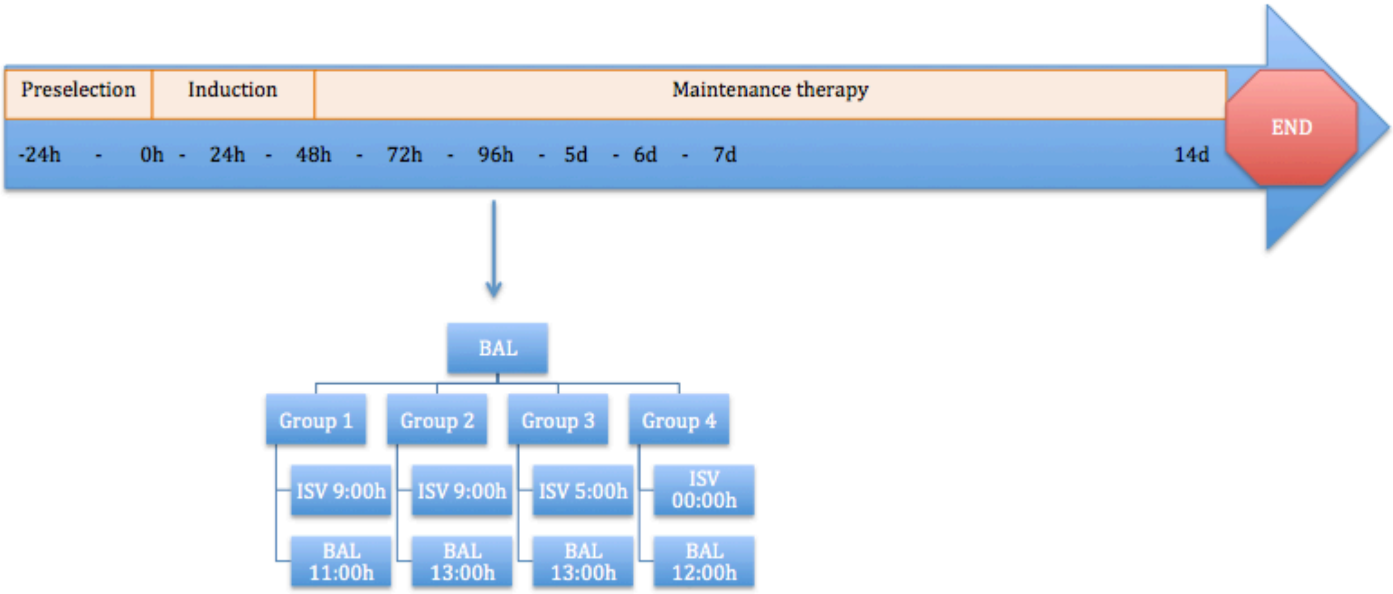

4.1.1 *Expected duration*

The duration of participation in the study will be until the end of follow-up, withdrawal of consent or loss to follow-up, whichever comes first. Participants will be monitored for adverse events whether they receive the complete treatment or not.

The duration of treatment with Isavuconazole is independent of the patient's participation in the study, since it will be maintained based on clinical practice. Patients will end the study 2 weeks after the start of treatment with isavuconazole. Overall, the maximum estimated duration for each participant is three weeks, comprising from the selection phase to the end of the follow-up. A total study duration of 18 months is expected.

4.2 Definition of end of study

The study will continue until the last eligible patient has completed the initial 14 days of treatment. The completion of the trial is expected to coincide with the last follow-up visit of the last patient included.

All follow-up completion of the study participants and the reasons for completion should be documented both in the medical record and in the clinical report form (CRF).

All participants have the right to withdraw their consent at any time, without any repercussion for them. In addition, treatment will be discontinued at the discretion of the investigator or the participant if any adverse effects occurr that justify it.

4.3 Criteria for premature completion of the study

The Sponsor reserves the right to finish the study at any time in the following cases:

- due to safety problems;
- due to problems with the supply of the drug under study;

- if compliance with the protocol is very poor or problems arise with the performance of the trial that may limit its ability to provide an answer to the main research question.

The decision based on these criteria will only be made once all the measures to improve the limiting problems have been exhausted.

If this is necessary, participants should be summoned as soon as possible for a visit and the same evaluations described in the End of Study Visit should be performed. The investigator may be informed of the additional procedures that must be followed to ensure that appropriate consideration is provided for the protection of the interests of the participants.

If the study is interrupted prematurely, the Sponsor will immediately inform the investigators, the corresponding Research Ethics Committee (REC) and the Competent Authorities, as appropriate, and specify the reasons for the interruption or closure. The Investigator will inform the study participants.

5 PATIENTS SELECTION

5.1 Study population

A total of 12 patients, over 18 years of age, lung transplant recipients with IFD diagnosis and indication for isavuconazole treatment according to the Summary of Product Characteristics will be included. The investigator or his designee should ensure that only patients who meet all of the following inclusion criteria and none of exclusion will be offered their participation in the study. Written informed consent must be obtained before any selection procedure, no specific procedure of the protocol should be performed, until the subject has signed and dated the informed consent document approved by the REC .

All data on the inclusion / exclusion criteria must be verifiable in the participant's source documents.

Patients included in this study may not participate in parallel studies of drugs or devices under investigation.

5.2 Selection criteria

5.2.1 Inclusion criteria

Patients eligible for inclusion in this study must meet all of the following criteria:

- Receipient of a lung transplant, older than 18 years of age , with indication of treatment with isavuconazole according to the Summary of Product Characteristics.

- Intent and ability to meet scheduled visits, treatment plan, laboratory analysis and other study procedures.
- They must be legally competent and able to understand, sign and date the informed consent form.

- Signing of the written informed consent in accordance with IHC / GCP and local legislation, obtained before any study procedure.

5.2.2 Exclusion criteria

Patients eligible for inclusion in this study should not meet any of the following exclusion criteria:

- Allergy or intolerance to isavuconazole.
- Contraindication for bronchoscopy and / or BAL.
- Any clinical condition, and / or analytical disorder that, in the opinion of the investigator, is considered clinically relevant to participate in the study.
- Individuals who show inability to follow the instructions or to collaborate during the study.
- Women with positive results of the pregnancy or undergoing breastfeeding period.
- Having participated in another clinical trial during the 3 months prior to the start of the study in which a research drug or a commercially available drug was tested.
- Lack of intent or inability to follow the procedures described in the protocol.
- Inability to grant written informed consent.

5.3 Selection failures

The concept of failure or selection failure refers to those cases in which the subject who has already signed the Informed Consent Document drops out or is withdrawn from the study before administering the first dose of the treatment under study.

Whenever a patient who has already given his informed consent to participate in the study does not meet the requirements of the inclusion and exclusion criteria mentioned above and bronchoscopy has not been performed (for the determination of isavuconazole levels at the bronchopulmonary level), he/she should discontinue the study and it will be considered a selection failure (which, in addition, will not be counted towards the sample size).

5.4 Drop-out or withdrawal of the subject

“Drop-out” cases are those in which the participation of a subject is completely lost - including safety monitoring - without him/her having withdrawn their consent.

5.4.1 Criteria for definitive discontinuation of the drug under study

The administration of isavuconazole will be carried out according to clinical practice, the interruption of treatment will be linked to the medical decision, outside the study.

5.4.2 Withdrawal from the study

5.4.2.1 Withdrawal of consent to participate in the study

As stated in the Declaration of Helsinki (Fortaleza, Brazil October 2013) and established in current legislation, participants have the right to withdraw their consent to participate in the trial at any time without justifying the decision and without implying damage to your medical care. However, this implies that no new information about them will be collected or processed nor will any new intervention be carried out for the study.

If a participant wishes to withdraw their consent, the investigator must explain the difference between withdrawing from the treatment and the entire trial, so that the decision is made knowingly.

5.4.2.2 Loss of subjects during follow-up

Given the underlying characteristics of the research question, the inclusion of 12 patients who receive the drug under study and who undergo the bronchoscopy planned on day 4 of treatment is required for the analysis. Consequently, the premature withdrawal of a trial participant will involve recruiting a new patient. Measures to control the withdrawal rate in order to avoid extendidng the trial due to these replacements, include a careful selection of participants, an adequate explanation of the requirements and procedures of the trial before inclusion and the explanation of the consequences of an early withdrawal.

The decision and the reason for the not performaning the bronchoscopy should be documented in the patient's medical history and in the CRF. When the reason is unknown, reasonable efforts will be made to find out (if a patient is lost during follow-up, they should try to contact him), always taking into account and respecting that the participants may decide to withdraw from the trial at any time, for any reason and without the need to justify your decision. That the patient does not provide the reason for his withdrawal or when this is unknown, does not exempt from registering this absence in the CRF.

6 TREATMENT

6.1 Assignment of subjects to treatments and masking

6.1.1 Treatment groups

All participants included will receive the product under investigation: oral Isavuconazole at the doses and dosage regimen contained in the technical file:

- Loading dose: 200 mg / 8h the first 48 hours.

-Maintenance dose from day 3: 200 mg / 24h.

6.1.2 Treatment assignment method

Not applicable, the study is of a single branch of treatment with Isavuconazole.

6.1.3 Masking

This is an open study and masking is not applicable.

6.2 Research treatment

6.2.1 Presentation of the product under investigation

All participants included will receive the product under investigation: CRESEMBA®, 100 mg hard capsules (Isavuconazole). Each capsule contains 100 mg of isavuconazole (such as 186.3 mg of isavuconazonium sulfate.

Its active substance, Isavuconazole, is a drug that belongs to the azole family, specifically it is a triazole. Like the rest of the triazoles, their mechanism of action consists in the inhibition of the enzyme 14-alpha-lanosterol demethylase, an activity that results in a depletion of the ergosterol from the fungal cell membrane. Isavuconazole has a broad spectrum of antifungal activity with

high in vitro activity compared to most of Candida, Cryptococcus, and Aspergillus species; Isavuconazole has moderate activity against Mucorales. Isavuconazole has activity against other clinically relevant yeasts and filamentous fungi. Isavuconazole presents cross resistance with voriconazole in the case of Aspergillus fumigatus and cross resistance with the rest of triazoles in the case of Candida spp.

Isavuconazole (Cresemba®) has been authorized in adult patients for the treatment of:

- Invasive aspergillosis.
- Mucormycosis in patients for whom amphotericin B is not appropriate.

Isavuconazole is available as a powder for intravenous formulation, as well as in oral capsules, in both cases as isavuconazonium sulfate. The capsules contain 186.3 mg of isavuconazone sulfate equivalent to 100 mg of isavuconazole, and the vials come with 372.6 mg of isavuconazonium sulfate equivalent to 200 mg of isavuconazole. The recommended drug dosage, both for oral and intravenous formulation, consists of the administration of 6 loading doses (200 mg of isavuconazole / 8 h the first two days) followed by a maintenance dose of a single daily dose of 200 mg. The duration of treatment depends on the clinical response of the patient, although treatments lasting more than six months should be carefully evaluated.

6.2.2 Dose regimen

All participants included will receive the product under investigation: oral Isavuconazole at the doses and dosage regimen contained in the Summary of Product Characteristics:

-Dosis load: 200 mg / 8h 48 hours.

-Dosis maintenance from day 3: 200 mg / 24h.

6.2.3 Duration of treatment

The administration of isavuconazole will be carried out according to clinical practice, the duration of treatment will be linked to the medical decision, outside the study.

6.2.4 Treatment administration mode

Route of administration: Oral administration.

6.2.5 Preparation and dispensing of study medication.

There are no specific preparation instructions applicable to the drug under study.

The reception of the study medication will be registered at the Pharmacy Unit of the Hospital Universitario Puerta de Hierro-Majadahonda.

The investigational product will be dispensed from the hospital Pharmacy through the usual channels for hospitalized patients. The drug will be administered by the nursing staff of the Pneumology Unit of the Hospital Universitario Puerta de Hierro-Majadahonda.

6.2.6 Labeling and packaging of study medication

This trial is considered to be of a low level intervention trial, according to Royal Decree 1090/2015, of December 4, since the drugs studied are authorized and commercialized, the drugs under investigation are used in accordance with the terms of the marketing authorization. In these cases, the legislation exempts from the need for re-labeling or reconditioning. The

dispensing of the drug will be carried out following the usual channels for for hospitalized patients, as established in the Center.

6.2.7 Supply and storage of medication

This trial is considered to be of a low level intervention trial, according to Royal Decree 1090/2015, of December 4, since the drugs studied are authorized and commercialized, the drugs under investigation are used in accordance with the terms of the marketing authorization. Isavuconazole will be supplied by the participating Center Pharmacy, following the channels for the supply of medication to hospitalized patients

The study medication does not require special storage conditions.

6.3 Prior and concomitant treatments

6.3.1 Prior medication

All medications received by patients in the week prior to the start of the study treatment, including over-the-counter products, natural food supplements, vitamins and medicinal plants, will be recorded as prior medications in the CRF.

6.3.2 Concomitant treatment allowed

There is no restriction regarding the use of medications and concomitant therapies.

All medications (except study medication) and non-drug therapies administered during the study should be listed in the corresponding section of the concomitant medication CRF.

The Sponsor declares that the use of any concomitant treatment will be carried out according to the authorized conditions of use or, failing that, according to the usual clinical practice.

6.3.3 Prohibited concomitant therapy

There is no restriction regarding the use of medications and concomitant therapies.

6.4 Treatment accountability and compliance with study medication

6.4.1 Method for the evaluation of therapeutic compliance

All doses administered and delivered to the patient will be recorded in the CRF.

7 STUDY PROCEDURES

7.1 Calendar of visits and study flow

Table 1 lists all the evaluations applicable to each phase of the study and indicates with an “X” the visits in which they must be carried out. All data obtained from these evaluations must be confirmed in the patient's source documentation. All evaluations must be carried out within the time periods specified for each visit.

Visit number	VISIT 0 (SCREENING)	VISIT 1 (TREATMENT INITIATION)	VISIT 2	VISIT 3 (BRONCHOSCOPY)	VISIT 4	END OF STUDY VISIT	EARLY DISCONTINUATION VISIT
Day	-1 – 0 days	24h	4d	5d	7d	14d	-
Informed Consent	X						
Inclusion/exclusion criteria	X						
Demographics	X						
Clinic history	X						
Physical examination	X					X	X
Vital signs	X					X	X
Electrocardiogram	X1					X	X
Biochemistry	X2					X	X
Blood count	X3					X	X
Coagulation	X4					X	X
Determination of tacrolimus levels 5		X	X	X	X		
Determination of mycophenolate levels		X	X	X	X		
Bronchoalveolar lavage (BAL)				Xi			
Determination of plasma isavuconazole6			X	X	X		
Adverse events		X	X	X	X	X	X
Concomitant medications	X	X	X	X	X	X	X
Isavuconazole administration7		X	X	X	X		

1 The electrocardiogram of the selection visit will be valid both on the day of the selection and on the previous 7 days.

2 The biochemistry of the selection visit will be valid both on the day of the selection and on the previous 7 days.

3 The blood count of the selection visit will be valid both on the day of the selection and on the previous 7 days.

4 Coagulation of the selection visit will be valid both on the day of the selection and on the previous 7 days.

5 Analytical extraction for the determination of tacrolimus levels should be performed just before the administration of Tacrolimus. Tacrolimus should be administered at least one hour before the administration of food and other drugs taken by the patient.

6 In group 1, if the administration of Isavuconazole is performed at 9:00 a.m., bronchoalveolar lavage will be performed at 11:00 a.m. In group 2, if the administration of Isavuconazole is performed at 9:00 a.m., bronchoalveolar lavage will be performed at 1:00 p.m. In group 3, the administration of Isavuconazole will be performed at 5:00 a.m. so that bronchoalveolar lavage is performed at 1:00 p.m. On that day, the dose of tacrolimus will also be administered at 8: 00h (since tacrolimus can be administered one hour before or 2-3 hours after the administration of other drugs, to obtain the maximum possible absorption of tacrolimus). In group 4, the administration of Isavuconazole will be carried out at 00:00, so that bronchoalveolar lavage is performed at 12: 00h. At visit 3, determination of plasma Isavuconazole levels will be performed before the last administration of the drug and coinciding with the time of bronchoalveolar lavage.

7 Isavuconazole will be administered according to the data sheet: 200mg / 8h during the first 48 hours. Later 200mg / 24 hours. The first dose in the morning will be administered one hour after the administration of tacrolimus.

7.2 Selection (days –1 to 0 days)

7.2.1 Summary of the selection period

Lung transplant recipients with invasive fungal disease will be selected.

Before carrying out any evaluation or study procedure, informed written consent must be obtained.

During the selection visit the inclusion and exclusion criteria will be evaluated. All documentation related to the results of the tests performed to determine eligibility is required to be archived as a source document.

7.2.2 Selection procedures

During the selection period, the following procedures and / or evaluations will be carried out:

- Obtaining signed and dated informed consent;
- Review of demographic data, medical history and current medical conditions, including those regarding the selection criteria: toxic habits, history of allergies.
- Registration of all pharmacological and non-pharmacological treatments, including over-the-counter products, natural food supplements, vitamins and medicinal plants in the previous week. Information that will be collected on previous or concomitant medications includes: tradename, reason for treatment, start date, end date and if continued, dose regimen.
- Full physical examination, including weight, height, blood pressure, heart rate, respiratory rate and temperature.
- 12 lead electrocardiogram (it will be valid both on the day of the selection and on the previous 7 days).
- Analytical tests (they will be valid both on the day of the selection and in the previous 7 days).
    - o Hemogram (red blood cells, hemoglobin, hematocrit, MCV, MCH, MCHC, platelets, MPV, leukocytes, neutrophils, eosinophils, basophils, lymphocytes, monocytes);
    - o Coagulation (prothrombin time, APTT, fibrinogen); Y
    - o General biochemistry with liver profile (Na +, K +, Cl-, Magnesium, glucose, urea, creatinine, albumin, total bilirubin, AST, ALT, GGT, Lactate dehydrogenase and alkaline phosphatase).

7.3 Induction period and treatment maintenance period (day 1 to 14)

7.3.1 Summary of the treatment period

During this phase each patient will receive Isavuconazole according to the data sheet (200mg every 8 hours for 48 hours and, later, 200mg every 24 hours).

7.3.2 Procedures during treatment visits

During the induction phase and the maintenance period of the treatment, patients will undergo the following evaluations:

<u>Visit 1 (First day of treatment)</u>

- Determination of plasma levels of tacrolimus. Analytical extraction for the determination of tacrolimus levels should be performed just before the administration of Tacrolimus. Tacrolimus should be administered at least one hour before the administration of food and other drugs taken by the patient.
- Determination of mycophenolate plasma levels. Analytical extraction for the determination of mycophenolate levels should be performed just before the administration of mycophenolate.
- Recording of all adverse events.

- Recording of all concomitant medication.
- Administration of Isavuconazole.

Visit 2 (72 hours after the start of treatment)

- Determination of the plasma levels of Isavuconazole.
- Determination of plasma levels of tacrolimus. Analytical extraction for the determination of tacrolimus levels should be performed just before the administration of Tacrolimus. Tacrolimus should be administered at least one hour before the administration of food and other drugs taken by the patient.
- Determination of mycophenolate plasma levels. Analytical extraction for the determination of mycophenolate levels should be performed just before the administration of mycophenolate.
- Registration of all adverse events.
- Registration of all concomitant medication.
- Administration of Isavuconazole.

Visit 3 (96 hours after the start of treatment)

- Determination of plasma levels of tacrolimus. Analytical extraction for the determination of tacrolimus levels should be performed just before the administration of Tacrolimus. Tacrolimus should be administered at least one hour before the administration of food and other drugs taken by the patient.
- Determination of mycophenolate plasma levels. Analytical extraction for the determination of mycophenolate levels should be performed just before the administration of mycophenolate.
- BAL bronchoscopy to determine levels of Isavuconazole at the bronchopulmonary level. The timing of bronchoscopy will depend on the group to which the patient has been assigned:
    - o In group 1, if the administration of Isavuconazole is performed at 9:00 a.m., bronchoalveolar lavage will be performed at 11:00 a.m.
    - o In group 2, if the administration of Isavuconazole is performed at 9:00 a.m., bronchoalveolar lavage will be performed at 1:00 p.m.
    - o In group 3, the administration of Isavuconazole will be performed at 5:00 a.m. so that bronchoalveolar lavage is performed at 1:00 p.m. That day, the dose of tacrolimus will also be administered at 8:00 a.m.
    - o In group 4, the administration of Isavuconazole will be carried out at 00:00, so that bronchoalveolar lavage is performed at 12: 00h.
- Determination of plasma levels of Isavuconazole before drug administration.
- Determination of the plasma levels of Isavuconazole concomitantly with bronchoscopy for BAL.
- Registration of all adverse events
- Registration of all concomitant medication.
- Administration of isavuconazole.

Visit 4 (7 days after the start of treatment)

- Determination of plasma levels of tacrolimus. Analytical extraction for the determination of tacrolimus levels should be performed just before the administration of Tacrolimus. Tacrolimus should be administered at least one hour before the administration of food and other drugs taken by the patient.
- Determination of mycophenolate plasma levels. Analytical extraction for the determination of mycophenolate levels should be performed just before the administration of mycophenolate.
- Determination of the plasma levels of Isavuconazole.
- Registration of all adverse events.
- Record of all concomitant medication.
- Administration of Isavuconazole.

End of study visit (14 days after the start of treatment)

- Full physical examination, including weight, height, BP, FC, FR and AT.
- 12-lead electrocardiogram.
- Analytical tests:
  - o Hemogram (red blood cells, hemoglobin, hematocrit, ACV, MCH, ACHC, platelets, APV, leukocytes, neutrophils, eosinophils, basophils, lymphocytes, monocytes);
  - o Coagulation (prothrombin time, APTT, fibrinogen); Y
  - o General biochemistry with liver profile (Na +, K +, Cl-, Magnesium, glucose, urea, creatinine, Albumine, total bilirubin, AST, ALT, GGT, LDH and alkaline phosphatase).
- Record of all adverse events.
- Record of all concomitant medication.

Early withdrawal visit

- Full physical examination, including weight, height, BP, FC, FR and AT.
- 12 lead electrocardiogram.
- Analytical tests:
  - o blood count (erythrocytes, hemoglobin, hematocrit, MCV, MCH, MCHC, platelets, APV, leukocytes, neutrophils, eosinophils, basophils, lymphocytes, monocytes);
  - o coagulation (prothrombin time, APTT, fibrinogen); Y
  - o General biochemistry with liver profile (Na +, K +, Cl-, Magnesium, glucose, urea, creatinine, Albumine, total bilirubin, AST, ALT, GGT, LDH and alkaline phosphatase).
- Record of all adverse events.
- Registration of all concomitant medication.

8 PHARMACOVIGILANCE

8.1 Collection and notification of adverse events

From the signing of the Informed Consent and until the end of the study, all adverse events (AE) that the investigator considers related to the study procedures will be recorded.

As part of the medical history, health problems (including clinically significant vital analytical values / constants located outside the reference range) that were diagnosed or known before the signing of informed consent will be recorded.

All the analytical results, the vital signs and the results or findings of the systemic physical examination and the ECG should be evaluated by the Investigator in order to determine their clinical importance. The analytical results, the findings of vital signs and the findings of the ECG abnormal isolated (ie not part of a notified diagnosis) should be reported as an adverse event are symptomatic, cause interruption of the administration of the study drug, requiring corrective treatment or constitute an adverse event according to the clinical judgment of the investigator.

At each visit, the investigator will determine whether adverse events have occurred through direct observation, spontaneous notification by participants or direct question to participants. Subjects should be asked in general, without asking about the appearance of specific symptoms. The investigator must evaluate all AE to determine its severity, intensity and causality according to the definitions in the section.

Investigator's evaluation must be clearly documented in the original documentation of the study center with the signature of the investigator.

The diagnosis must be notified by the term of AE or the SAE (serious adverse event). If no diagnosis is available, the primary sign or symptoms will be notified by the term AE or SAE, including additional details in the description until a diagnosis is available. If the signs or symptoms are different and do not indicate a common diagnosis, they should be reported as individual AE or SAE entries.

In case of severe AE due to hospitalization, the reason for hospitalization will be reported as the SAE (diagnosis or symptom that requires hospitalization).

The investigator should follow up the subjects with AE until the adverse event is resolved. In the case of unresolved AE, including abnormal and significant analytical values in the end-of-study evaluation, these events will be monitored until they are resolved or until they are no longer clinically relevant.

8.2 Adverse event

8.2.1 Definitions

- **Adverse event (AE)** is any incident that is detrimental to health in a patient or clinical trial subject treated with a medication, even if it does not necessarily have a causal relationship with that treatment.

- Adverse drug reaction (ADR) is any harmful and unintended reaction to a drug under investigation, regardless of the dose administered.

- **Serious Adverse Event (SAE)** and **Serious Adverse Reaction (SAR)** is any adverse event or adverse reaction that, at any dose: causes death, threatens the life of the subject, necessitates hospitalization or prolongation of the subject, causes disability or disability permanent or important, or

give rise to a congenital anomaly or malformation. For the purposes of notification, suspicions of adverse events or adverse reactions that are considered medically important will be treated as serious, even if they do not meet the above criteria. An example of these events is allergic bronchospasm that requires intensive treatment in an emergency service or at the home of the subject, blood dyscrasias or seizures that do not require hospitalization, or the development of dependence or abuse of the drug.

The medical and scientific criteria should be used to decide whether other situations that have not resulted in the outcomes listed in the previous definitions should be reported as SAE.

The threat of life is understood as the situation in which, in the opinion of the doctor, had the patient died without a timely therapeutic intervention.

The following situations will not meet the SAE criteria:

-In the event that hospitalization or its prolongation is necessary to perform a procedure required by the protocol (for example, if there will be day or night visits for biopsies or surgeries required by the protocol).

-In the event that hospitalization or its prolongation is part of the routine procedure of the center.

-In case of hospitalization scheduled by a preexisting process that has not worsened.

Unexpected adverse reaction (UAR) is that adverse reaction whose nature or severity does not correspond to the information regarding the product (in this case, since Isavuconazole is a marketed drug, the product data sheet).

Serious and unexpected adverse reaction (SUSAR) is that adverse reaction in which both characteristics converge; On the one hand, it is serious, that is, that at any dose, it causes death, threatens the life of the subject, causes disability or permanent or significant disability, or gives rise to a congenital anomaly or malformation and is also unexpected, that is, , its nature or severity does not correspond to the information regarding the product available.

8.2.2 Intensity evaluation

The intensity of an AE will be classified according to the WHO toxicity scale (recommendations for the classification of acute and subacute toxic effects). AEs not listed in the table will be classified according to the following scale:

- Mild: banal adverse events, of little importance and short duration, which do not substantially affect the participant's life. (Grade I)
- Moderate: adverse events that cause enough discomfort to interfere with the normal life of the participant. (Grade II)
- Intense: adverse events that imply an inability to work or perform the participant's usual activity. (Grade III)
- Very intense: life-threatening adverse events (Grade IV)

Intensity versus seriousness. The term “intensity” is used to describe the intensity of a specific event, although the event itself may have a relatively small medical importance. It is not the same as “seriousness”, which is based on the outcome of the event in the participant at the time of the event.

8.2.3 8.2.3 Causality assessment

The causal relationship between the product under investigation and the appearance of AE / SAE will be established, based on a clinical trial. For this, other causes will be considered and studied, such as the natural history of the underlying diseases, concomitant treatment, other risk factors and the temporal

relationship of the event with the product under investigation. In addition, the Investigator's Brochure ofor the product under investigation will be consulted.

The causal relationship of an AE / SAE with the medication under study will be evaluated taking into account the administration of Isavuconazole according to the investigator's judgment and the following definitions. The causality assessment should be performed according to the available information and can be updated as new information emerges.

Unlikely relationship: the adverse event does not occur after a plausible chronological sequence related to the administration of the product under study and / or is reasonably explainable by other factors, such as the participant's clinical status or other concomitant therapeutic, toxic or environmental interventions. In addition, it does not match the known or expected response pattern of the drug.

Possible relationship: the adverse event occurs after a plausible chronological sequence related to the administration of the product under study, but can also be explained by the clinical status of the participant or other concomitant therapeutic, toxic or environmental interventions. In addition, it matches the known or expected response pattern of the drug.

Likely relationship: the adverse event occurs after a plausible chronological sequence related to the administration of the product under study, cannot be reasonably explained by the participant's clinical status or other concomitant therapeutic, toxic or environmental interventions, and after withdrawal or decrease of the dose of the suspect drug the event follows a logical clinical sequence. In addition, it matches the known or expected response pattern of the drug.

Very likely relationship: the adverse event occurs after a plausible chronological sequence related to the administration of the product under study, cannot be reasonably explained by the participant's clinical status or other concomitant therapeutic, toxic or environmental interventions, after withdrawal or decrease from the dose of the suspect drug the event follows a logical clinical sequence, and it is necessary that after the re-administration of the suspicious drug the adverse event reappears. In addition, it matches the known or expected response pattern of the drug.

No relationship: adverse event clearly due to causes beyond the medication under study, and the criteria of another causal relationship are not met.

Not assessable relationship: any notification that suggests an adverse effect, which cannot be judged because the information is insufficient or contradictory, and that cannot be complemented or verified.

8.2.4 Measure adopted in relation to the drug under study

Given the characteristics of the study, the withdrawal of the drug under study is only foreseen when it is necessary to adopt a measure with the drug under study after the appearance of an AE / SAE , which will be at the discretion of the medical team.

Withdrawal of the drug. Definitive interruption of the administration of Isavuconazole.

8.2.5 Other measures taken for the event

- None. No action was required
- Need for medication. Medication with / without a prescription was required to treat AE / SAE.

8.2.6 Outcome of the adverse event

The options are as follows:

- Recovered or resolved. The subject recovered completely from AE with no observed residual effect.
- In the process of recovery or resolution. The AE improved, but it has not been fully resolved.
- Not recovered or not resolved. The AE is still present and is observable.
- Recovered or resolved with sequels. The residual effects of AE are still present and observable.
- Mortal, should be considered if death is a direct result of AE.
- Unknown.

8.3 Safety notifications - Procedure for Investigators

The AEs will be collected at each visit from the participant's careful clinical observation, laboratory analysis, spontaneous communication from the participant and also through an open interrogation by the investigator.

All AE (serious or not) that occur during the course of the study should be detailed in the medical history and reflected in the CRF. The investigator will also decide if the adverse event is, according to his criteria, related or not to the drug under study - decision that should also be reflected in clinical history and CRF.

At each visit, all the AE that the participant has submitted since the previous visit must be registered in the CRF-specific Adverse Event Form.

All serious adverse events occurring during the trial must be reported to the Sponsor immediately.

When appropriate (in case of serious and unexpected adverse reactions), the Sponsor will notify the Spanish Medicines Agency and REC about the event in question by means of the corresponding notification sheet (Annex 1: Notification Form of Serious and Unexpected Adverse Reactions), in a maximum period of 15 calendar days.

The principal investigator will immediately notify the Sponsor or Delegated Center of all serious adverse events regardless of their degree of causality with the drug under study.

PV Manager: Unidad de Farmacovigilancia. Hospital Universitario Puerta de Hierro Majadahonda
Phone (0034) 911917070,
Fax (0034) 911917650,
e-mail farmacologia_clinica@idiphim.org

The notification period for SAE will be 24 hours from the moment the investigator has had knowledge of it for those events that have caused the death of the subject, or is life- threatening. For the rest of the SAE, the notification period will be 48 hours so that the Sponsor can fulfill its regulatory obligations.

The initial communication of the SAEs must be written and using the CRF Serious Adverse Event Notification Form. The form must be sent via fax to the number indicated on the same page.

In the case of SAE, the Investigator must provide the Sponsor with all the documentation related to the event (additional laboratory tests, discharge reports, etc.).

The investigator must also monitor the SAE and notify in the same way the information related to the event until it has been sent, returned to its baseline situation or, in case of permanent affectation, until the process is stabilized.

In case of death, the Investigator must provide the Sponsor and the REC involved all the additional information requested.

8.4 Procedures for the immediate notification of serious and unexpected adverse reactions by the Sponsor

The Sponsor will notify those events that are serious and unexpected that may be related to the treatments under investigation (that is, those serious and unexpected adverse reactions, SUSAR) to the Spanish Medicines Agency, to the competent organs of the Autonomous community involved. These records will be submitted to the Spanish Medicines Agency when requested.

The notification will be made through the Adverse Reaction Suspicion Notification Form attached as Annex (annex 1): SUSAR Notification Form.

Information on adverse events that are not serious or unexpected and those that are considered unrelated to the treatments under study will be collected in tabular form at the end of the clinical trial or coinciding with the intermediate analyzes when they were planned.

The Sponsor will keep detailed records of all the AEs that are communicated by the Investigators.

The Sponsor of the study will notify the competent authorities of any information that could modify the risk / benefit ratio of the investigational medicinal product, or determine changes in its administration schedule or in the conduct of the test, for example:

- A qualitative change or an increase in the percentage of occurrence of the expected SAR, which is considered clinically important.
- SUSAR that occur after the completion of a clinical trial and that are notified by the Investigator to the Sponsor.
- New events related to the conduct of the trial or the development of the investigational medication and that probably affect the safety of the subjects, such as:
    - o Serious adverse events that may be associated with the test procedures and may modify its performance
    - o SARs related only to a non-investigational drug (MNI) that are considered relevant since they are not subject to the general rules for expedited notification of individual SUSAR cases.
- Any recommendation, which is relevant for the safety of the subjects.

The maximum period of notification will be 15 calendar days from the moment the Sponsor has been aware of the suspicion of RA. Those SUSAR that have caused the death of the subject, or endangered his life, will have a maximum notification period of 7 calendar days from the moment the Sponsor has had knowledge of it. This information will be completed, when possible, during the following 8 days. In addition, if additional information is obtained, it must also be notified as quickly as possible.

8.5 Analytical tests

The determinations for hematology, blood biochemistry and coagulation will be analyzed.

8.6 Vital signs

Vital signs, including blood pressure, heart rate, respiratory rate and body temperature will be determined at the times specified in the study calendar.

8.7 Electrocardiograms

The 12 lead ECGs in the supine position will be obtained at the times specified in the procedure calendar.

8.8 Physical exams

A complete physical examination will be performed during the selection period and at the additional times specified in the procedures calendar. Whenever possible, it will be the same professional who performs all the examinations to identify variations with respect to baseline values. Clinically significant variations with respect to baseline values should be reported as AE.

The examinations directed by symptoms performed during the treatment period will be based on the clinical history of the subject and the AE and will include review of the anthropometric measures, review of systems and devices.

8.9 Other explorations

Not applicable

9 DATA COLLECTION AND MANAGEMENT

9.1 Confidentiality of data

The information on the participants will be kept confidential and will be handled under the applicable laws and regulations, Regulation (EU) 2016/679 of April 27 regarding the protection of natural persons in relation to the processing of their personal data and the Spanish regulations of application, Organic Law 3/2018, of December 5, on the Protection of Personal Data and guarantee of digital rights. Such regulation requires an authorization of the signed participant to inform the subject of the following:

- What protected health information (PHI) will be collected in the subjects of this study
- Who will have access to the information and why,
- Who will use or disclose such information,
- The rights of an investigation subject to revoke his authorization for his PHI to be used.

In the event that a patient revokes the authorization to collect or use PHI, the investigator retains the ability to use all the information collected before the revocation of the subject's authorization.

9.2 Center monitoring

Before starting the study, a review of the protocol and the CRF with the Investigators will be carried out at the start visit. During the study, the monitor will periodically visit the Center, to check that the participants' records are complete, the accuracy of the CRF data, adherence to the protocol and the Standards of Good Clinical Practice, recruitment progress and also to ensure that the study medication is being stored, dispensed and accounted for according to the established specifications. The staff responsible for the study should be available to assist the monitor during visits.

The investigator must keep the original documents of each patient who is participating in the study, which will consist of the medical history, including medical and demographic information, laboratory

data, ECGs and the results of any other test. All information that is recorded in the CRF must be verifiable with these source documents that are normally found in the patient's medical history. The investigator must also keep the original Informed Consent (a copy will be given to the patient).

The investigator must accept that the monitor review all relevant original documents to confirm that they are consistent with the entries of the CRF. A complete verification of the existence of informed consent will be carried out, that all inclusion / exclusion criteria and the documentation of the SAE are met. Additional checks of the consistency of the original data in the CRFs will be carried out, according to the specific monitoring plan for this study.

9.3 Data collection

A paper CRF will be used. For each patient included in the study, a Clinical Report Form or CRF will be completed. This also applies to patients who do not complete the full follow-up provided in the trial. Subjects will not be identified by name or initials in the CRF or in any trial document. The only acceptable identification that will appear on the CRF or other documents is the patient's unique identification number. The investigator will keep contact information for all the participants, so that if necessary he/she can get in touch with them quickly.

All CRF must be filled in by duly authorized personnel. The investigator will keep the records and data during the test in compliance with all current legal and regulatory provisions. All data must be backed by original documents at the test center. Any record or document used as a source of information will be retained for review by authorized representatives of the Sponsor or regulatory bodies.

9.4 Database management and quality control

A data management plan will be carried out before the start of the definition of the database in which the recording process will be detailed and the error and consistency controls that will be carried out on the recorded data. A dictionary of variables will be generated in which the correspondence between the data contained in the CRF and the variables of the database will be detailed, as well as the encodings used and the meaning of the recorded values. In case of inconsistencies or errors in the data, requests for clarification will be generated for Investigators to verify or correct, which will be treated in an equivalent manner to the CRFs. Access to the database will be restricted to the Data Manager (design, data entry and debugging) and to the personnel in charge of data transcription (data entry).

Prior to the final database declaration, a check of the consistency of the values of the inclusion / non-inclusion criteria, of the clinical evaluations, of the results of complementary examinations, of the dates of visit, of compliance, of the medication received, from adverse events, from information on dropouts and from the evaluation of skin tolerability.

A definitive database will be declared that will be registered with signature and date. Two copy-protected copies of it will be kept, and paper listings of the variables contained in the database will be generated for archiving. The definitive database will be used for statistical analysis.

10 STATISTICAL METHODS AND DATA ANALYSIS

The statistical analysis will be carried out following the principles specified in the International Conference on Harmonization (ICH) Topic E9 (CPMP / ICH / 363/96).

Before the database is declared closed, a detailed statistical analysis plan will be available. A summary of the general approach to statistical analysis is presented. No intermediate analyzes are planned.

The data will be represented as absolute frequencies and percentages in the case of qualitative variables. For the quantitative variables the mean and the median and the standard deviation (SD) or the median and the interquartile range (IQR: P25; P75) will be used, according to their distribution. In the case of ordinal variables, depending on the number of categories, absolute frequencies and% or the median and IQR will be used, depending on the number of categories.

10.1 Analysis groups

Given the characteristics of the study, it is a descriptive study, only two analysis groups are planned:

- The safety and tolerability analysis group will include all patients who have received at least one dose of isavuconazole.
- The pharmacokinetic analysis group (PK) will include all patients who underwent bronchoscopy.

10.2 Study population data: demographic characteristics and other selection characteristics

Demographic data and other data related to patient selection will be descriptively summarized for the PK analysis group and the safety / tolerability group.

The medical history will be coded using the latest version of the MedDRA medical dictionary and will be summarized by categories of organ, apparatus or system and preferred term.

Categorical data will be presented in the form of frequencies and percentages. For continuous data, descriptive statistics such as mean and median and standard deviation (SD) or median and interquartile range (IQR: P25; P75), minimum and maximum will be presented.

10.3 Treatments (study treatment, concomitant therapies, compliance)

All study medication data will be summarized using the safety / tolerability group.

The actual treatment time, the duration of exposure, and the actual daily dose will be summarized.

Concomitant medications and significant non-pharmacological therapies before and after the start of the study treatment will be summarized.

10.4 Main objective

The main objective of this study is to describe the pharmacokinetic profile of oral isavuconazole at the bronchopulmonary level through the degree of penetration in the epithelial lining fluid and the alveolar macrophages in patients receiving lung transplantation with a diagnosis of invasive fungal disease.

10.4.1 Variable

The main variable is the AUC of isavuconazole at the ELF level and in CAs.

10.4.2 1Statistical hypothesis, model and method of analysis

No inferential analysis is planned as there is no control group. A descriptive analysis will be carried out.

10.4.3 Handling of unavailable values / withdrawal

The existence of unavailable values for the main variable is not foreseen, since the restitution of premature withdrawals from the trial is envisaged by recruiting new patients until the number of patients planned for the study is completed (12 patients).

10.5 Secondary objectives

The secondary objectives are:

- Establish the relationship between the pulmonary and systemic exposure of Isavuconazole.
- To evaluate the potential pharmacokinetic interaction of Isavuconazole with immunosuppressive drugs (tacrolimus and mycophenolate). This interaction will be studied in the steady state of the drugs evaluated.
- Evaluate the safety and tolerability of Isavuconazole.

The safety analyzes will be based on the safety / tolerability analysis group. The baseline value collected in the selection visit will be used as the baseline value for the safety analysis.

Given the nature of the study, no inferential analysis is planned for safety data.

10.5.1 Interaction evaluation

Tacrolimus and mycophenolate concentrations will be determined before and after 72 hours, 96 hours and 7 days after the start of treatment with isavuconazole.

The percentage of patients who required dose adjustment of the immunosuppressive drug after the start of treatment with isavuconazole will be determined.

10.5.2 Analysis of Adverse Events

The incidence of AE arising after the treatment under study will be summarized by class of organic system and MedDRA preferred term, severity, type AE and relationship with the study treatment.

A list of AE data per subject will be provided in which they will include, among others, the literal term, the preferred term, the category of organ, apparatus or system, the degree of intensity and the relationship with the drug under study. A list of deaths, other SAE and other significant AE will be provided, including those that force the suspension of the drug under study.

10.5.3 Analysis of analytical tests

Descriptive statistics will be provided for the analytical results and variations with respect to the baseline value per scheduled evaluation moment, end of follow-up visit.

Abnormal laboratory results will be classified according to version 4.03 of the NCI CTCAE criteria, if applicable.

10.5.4 Analysis of vital signs

Descriptive statistics will be provided for vital signs determinations and variations with respect to the baseline value for each scheduled evaluation time, including the end of treatment and follow-up visit.

10.5.5 Electrocardiogram analysis

Descriptive statistics will be provided for the electrocardiographic parameters (PR, RR, QRS, QT and QT intervals corrected with the Bazett formula) and variations with respect to the baseline value per scheduled evaluation time (end of follow-up visit).

10.5.6 Analysis of physical examinations

Descriptive statistics will be provided for the findings of the physical examinations with respect to the baseline value per scheduled time of evaluation, end of follow-up visit.

10.6 Calculation of sample size

In total, the inclusion of 12 lung transplant recipients is expected.

The number of subjects has been estimated based on the objective and characteristics of the study and the drug under study. Given the descriptive nature of the study, it has been considered that with the exposure of 12 subjects sufficient information will be obtained to determine the pharmacokinetic profile of oral isavuconazole at the bronchopulmonary level.

Currently, there are no data in the scientific literature about the pulmonary disposition of isavuconazole. Similar studies have been conducted for other azoles, which includes a limited number of patients (8-20 patients) (17-20).

10.7 Statistical analysis process

The Statistical Analysis Plan will describe the statistical methods and definitions for the analysis of pharmacokinetic and safety data, as well as the methods followed for Summarize other information in this clinical trial, such as demographic data and initial characteristics, exposure to the drug under study, and previous and concomitant medications. The SAP will also include a description of how to deal with missing, unused and false data.

All statistical analyzes will be performed with SAS Version 9.0 or later (SAS Institute, Cary).

11 ETHICAL CONSIDERATIONS AND ADMINISTRATIVE PROCEDURES

11.1 Ethical and regulatory compliance

The test will be carried out in accordance with the principles emanating from the Declaration of Helsinki (last update October 2013 Fortaleza (Brazil), and in the Oviedo Convention of the Council of Europe of 1997, ratified in 1999 and according to current legal regulations (Royal Decree 1090/2015) and will begin once the approval of the REC, the authorization of the Spanish Medicines Agency, and the agreement of the Director of the Institution have been obtained.

11.2 Responsibilities of the Investigator

The Investigator's responsibilities are:

a. To Sign with the Sponsor the test protocol.

b. Thoroughly know the properties of investigational drugs.

c. Ensure that informed consent is collected in accordance with the provisions of this royal decree.

d. Collect, record and notify the data correctly and ensure its accuracy.

and. Follow the instructions regarding the communication of adverse events established in the protocol.

f. Immediately notify the Sponsor of serious breaches of the protocol.

g. Ensure that all persons involved will respect the confidentiality of any information about the subjects of the trial, as well as the protection of their personal data.

h. Regularly inform the REC about the progress of the test.

i. Co-responsible with the Sponsor of the elaboration of the final report of the essay, giving their agreement with their signature.

11.3 Informed consent procedures

Eligible patients may only participate in the study after providing written informed consent (IC) approved by the Research Ethics Committee.

Informed consent must be obtained before performing any specific study procedure. The process of obtaining informed consent should be documented in the patient's source documents (medical history). The date on which it is obtained in IC will be recorded in the patient's CRF.

The Sponsor will provide the Investigators, in a separate document, with the information sheet for the participating patient. This document will conform to the guidelines of the GCPs, the IHC and the regulatory requirements. Any change in HIP must obtain Research Committee approval.

Each candidate to participate in the essay will be given the HIP along with the signature sheet of the Informed Consent. This document will collect all relevant information about the clinical trial that the participant should know:

1) Background and objectives;

2) Methodology used;

3) Treatment to be administered and procedures to be performed;

4) Expected benefits for him and society;

5) Risks derived from the study and possible adverse events;

6) Voluntary nature of their participation, as well as the possibility of withdrawing from the study at any time, without the need to justify themselves and without causing damage to their attendance;

7) Place and time of conservation of the data / samples, people who will have access to the data of the volunteer and planned measures in the matter of personal data protection and confidentiality;

8) Mode of economic compensation and planned tax treatment;

9) Contact, administrative and trial quality data;

10) Communication of the results and other relevant information.

The Principal Investigator or his collaborators are responsible for informing the subject, answering their doubts and questions and will be their main contact in case of urgency.

In accordance with current regulations, the investigator will obtain the written informed consent of the patient. The consent form should be reviewed with the patient candidate for inclusion in the study, answering all questions about the trial raised by the volunteer.

In addition, patients are free to refuse to participate and may revoke their consent at any time, without having to give explanations and without deriving any responsibility or harm to it. Informed consent must be obtained before initiating specific tests and assessments of the study.

11.4 Study termination

The Sponsor reserves the right to suspend this study under the conditions specified in the clinical trial agreement. The specific conditions for the completion of the study are described in Section 4.3.

11.5 Publications of the study protocol and the results

The results of the study may be published in scientific journals or medical congresses, but always maintaining confidentiality, without revealing any personal data that can identify the participants.

The Investigators will publish the results whether they are positive or negative. The authorship will take into account the members of the study steering committee, participating Investigators and people responsible for the coordination, monitoring, data analysis and writing of the articles.

All authors must have:

- Contributed substantially to the concept, design and / or implementation (inclusion of subjects), or to the acquisition, analysis and interpretation of the data, and
- Written or critically reviewed the proposed clinical publication for important intellectual content, and
- Approved the final clinical publication proposed for the presentation, and
- Have a thorough knowledge of the analysis / implementation of the trial.

11.6 Dissemination of data or results

The encoded data can be transmitted to third parties and other countries, but in no case will they contain identifying information of the participants, such as name and surname, initials, address, social security number, etc. In the event that this assignment occurs, it will be for the same purposes of the study described or for use in scientific publications, and always maintaining their confidentiality in accordance with the provisions of current legislation.

11.7 Documentation of the study, conservation and management of documents related to the study

The participating Center will keep the appropriate clinical and research records for this trial, in compliance with section 4.9 of IHC E6 BPC and with the regulatory and institutional requirements for the protection of the confidentiality of the participants in the study.

The clinical trial master file will comply with the provisions of Articles 57 and 58 of Regulation (EU) No. 536/2014 of the European Parliament and of the Council of April 16, 2014. Its content must take into account the supplementary guidelines. in this regard published by the European Commission.

The Sponsor and the Investigator will keep the contents of the master file in paper or digital format of each clinical trial for at least twenty-five years after the end of the trial.

The content of the master file will be kept in such a way that it can be easily made available to the competent authorities, if requested.

The clinical history of the subject of the trial must be guarded in accordance with the provisions of Law 41/2002, of November 14, and according to the maximum period allowed by the hospital.

Any transfer of ownership of the content of the master file will be documented and the new owner will assume the responsibilities set forth in this article.

The Sponsor will name the people in his organization responsible for the files and access to them should be limited to the designated persons.

The supports used to preserve the essential documents will be, in general, in electronic format and must ensure that the documents remain complete and legible during the planned period of conservation and that they are available to the competent authorities in case they request them. Any modification of the records must be traceable, allowing to know the initial and corrected data, as well as the date and signature of the author.

This documentation must be archived, preferably grouped by protocols, in a place that guarantees the confidentiality of the information during the required archiving time.

In the event that there are open judicial proceedings, the essential documents will be retained until there is a firm judicial decision.

11.8 Confidentiality of study documents and participant records

The Sponsor of the study undertakes that the processing, communication, and transfer of personal data of all participating subjects will comply with the provisions of Regulation (EU) 2016/679 of the European Parliament and of the Council of 27 April 2016 of Data Protection (GDPR) and in Organic Law 3/2018, of December 5, on Protection of Personal Data and guarantee of digital rights. In accordance with the provisions set forth herein, the participant may exercise the rights of access, modification, opposition, cancellation of data, limit the processing of data that is incorrect, request a copy or transfer the data provided to a third party (portability) the study.

To exercise these rights, the participating subject may contact the principal investigator of the study or the Data Protection delegate of the center / institution participating in the study. You will also have the right to contact the Data Protection Agency if you are not satisfied.

In order to guarantee confidentiality, the data collected for the study will be identified by a code and only the doctor of the responsible study, its collaborators, the competent sanitary authorities, the Committee of Ethics of the Research with medicines and the personnel authorized by the Sponsor (study monitors, auditors) may relate such data with the participant or their medical history when it is necessary to check data and study procedures.

11.9 Inspections and audits

The source data / documents must be available for audits requested by the Sponsor and inspections of regulatory agencies.

12 FUNDING AND INSURANCE

12.1 Civil Liability Insurance

This trial of low level of intervention is considered and therefore will not need to be covered by an insurance contract or financial guarantee since it is covered by the Servicio Madrileño de Salud (SERMAS) liability insurance, under Article 39.4 of the Royal Decree 1090/2015.

12.2 Economic aspects

12.2.1 Economic aspects

Before starting the study, the principal investigator, the institution and the Sponsor of the study will sign a clinical study contract. Said contract shall include the economic information agreed between the parties.

12.2.2 Economic compensation to participants

Given the nature of the study, participating patients will not get any benefit from their participation in the study.

13 ADHESION TO THE PROTOCOL

It is established that investigators apply due diligence to avoid deviations from the protocol. All significant deviations from the protocol will be recorded and reported in the final clinical trial report.

13.1 Modifications to the protocol

Any change or addition to this protocol may only be made in a written modification to the protocol that must be approved by the Sponsor, by the Health Authorities.

14 REFERENCES

1. Solé A, Ussetti P. Mold infections in lung transplants. Rev Iberoam Micol. 2014;31:229-36.
2. Kennedy CC, Razonable RR. Fungal Infections After Lung Transplantation. Clin Chest Med. 2017;38:511-520.
3. Pilarczyk K, Haake N, Heckmann J, Carstens H, Haneya A, Cremer J, Jakob H,Pizanis N, Kamler M. Is universal antifungal prophylaxis mandatory in adults after lung transplantation? A review and meta-analysis of observational studies. Clin Transplant.2016;30:1522-1531.
4. Husain S, Bhaskaran A, Rotstein C, Li Y, Bhimji A, Pavan R, Kumar D, Humar A, Keshavjee S, Singer LG. A strategy for prevention of fungal infections in lung transplantation: Role of bronchoalveolar lavage fluid galactomannan and fungal culture. J Heart Lung Transplant. 2018;16.pii: S1053-2498(18)31356-1.
5. Ullmann AJ, Aguado JM, Arikan-Akdagli S, Denning DW, Groll AH, Lagrou K,et al.Diagnosis and management of Aspergillus diseases: executive summary of the 2017 ESCMID-ECMM-ERS guideline. Clin Microbiol Infect. 2018;24 Suppl 1:e1-e38.
6. Patterson TF, Thompson GR 3rd, Denning DW, Fishman JA, Hadley S, Herbrecht R, Kontoyiannis DP, Marr KA, Morrison VA, Nguyen MH, Segal BH, Steinbach WJ, Stevens DA, Walsh TJ, Wingard JR, Young JA, Bennett JE. Practice Guidelines for the Diagnosis and Management of Aspergillosis: 2016 Update by the Infectious Diseases Society of America. Clin Infect Dis. 2016;63:e1-e60.
7. Maertens JA, Raad II, Marr KA, Patterson TF, Kontoyiannis DP, Cornely OA, Bow EJ, et al. Isavuconazole versus voriconazole for primary treatment of invasive mould disease caused by Aspergillus and other filamentous fungi (SECURE): a phase 3, randomized controlled,noninferiority trial. Lancet 2016;387:760-9.
8. Silva JT, Torre-Cisneros J, Aguado JM. Aspergilosis invasora en el receptor de trasplante sólido. Revista Iberoamericana de Micología 2018; 35 (4): 206-209.
9. Groll AH, Desai A, Han D, Howieson C, Kato K, Akhtar S, Kowalski D, Lademacher C, Lewis W, Pearlman H, Mandarino D, Yamazaki T, Townsend R. Pharmacokinetic Assessment of Drug-Drug Interactions of Isavuconazole With the Immunosuppressants Cyclosporine, Mycophenolic Acid, Prednisolone, Sirolimus, and Tacrolimus in Healthy Adults. Clin Pharmacol Drug Dev. 2017;6:76- 85.
10. McCarthy MW, Moriyama B, Petraitine R, Walsh TJ. Clinical Pharmacokinetics and Pharmacodynamics of Isavuconazole. Clinical Pharmacokinetics. 2018.
11. Rivosecchi RM, Clancy CJ, Shields RK, Ensor CR, Shullo MA, Falcione BA, Venkataramanan R, Nguyen MH. Effects of Isavuconazole on the Plasma Concentrations of Tacrolimus among Solid-Organ Transplant Patients. Antimicrob Agents Chemother. 2017;24;61(9).
12. Felton T, Troke PF, Hope WW. Tissue penetration of antifungal agents. Clin Microbiol Rev. 2014;27:68-88.

13. Petraitis V, Petraitiene R, Patriss WM, Strauss GE, Katragou A, Kovanda LL. Pharmacokinetcs and Concentration-Dependent Efficacy of Isavuconazole for Treatment of Experimental Invasive Pulmonary Aspergillosis. Antimicrobial Agents and Chemotherapy. 2016; 60 (5): 2718-2726.
14. Ter Avest M, Veringa A, van den Heuvel ER, Kosterink JG, Schölvinck EH, Tissing WJ, Alffenaar JC. The effect of inflammation on voriconazole trough concentrations in children. Br J Clin Pharmacol. 2017;83:678-680.
15. Kabulski GM, MacVane SH. Isavuconazole pharmacokinetcs in a patient ith cystic fibrosis following bilaterl orthotopic lung transplatation. Trasplantation Infectious Disease. 2018; e12878.
16. Rennard SI, Basset G, Lecossier D, O'Donnell KM, Pinkston P, Martin PG, Crystal RG. Estimation of volume of epithelial lining fluid recovered by lavage using urea as marker of dilution. J Appl Physiol. 1985. 1986;60:532-8.
17. Crandon JL, Benevicius MA, Fang AF et al. Bronchopulmonary disposition of intravenous voriconazole and anidalafungin given in combination to healthy adults. Antimicrob Agents Chemother. 2009; 53: 5102-7.
18. Capitano B, Potoski BA, Husain S et al. Intrapulmonary penetration of voriconazole in patients receiving an oral prophylactic regimen. Antimicrob Agents Chemother. 2006; 50: 1878-80.
19. Conte Jr JE, Golden JA, Krishna G et al. Intrapulmonary pharmacokinetics and pharmacodynamics of posaconazole at steady state in healthy subjects. Antimicrob Agents Chemoter. 2009; 53: 703-7.
20. Conte Jr JE, DeVoe C, Little E et al. Steady-state intrapulmonary pharmacokinetics and pharmacodynamics of posaconazole in lung transplant recipients. Antimicrob Agents Chemother. 2010; 54: 3609-13.
