# Supplementary material for: Bronchopulmonary penetration of isavuconazole in lung transplant recipients
Source: Antimicrob Agents Chemother. 2023 Oct 3;67(10):e00613-23. doi: 10.1128/aac.00613-23 (PMC10583689; doi:10.1128/aac.00613-23)
Supplement: Table S1 — Concentrations for each patient. [file aac.00613-23-s0002.docx]

**Supplementary Appendix Material**

Supplementary table. Concentrations for each patient

| **Patient** | **Timing of sampling after the last dose (h)** | **Serum concentration of ISA (µg/mL)** | | | | **ELF (µg/mL)** | | **ELF/Serum ratio** | |
| --- | --- | --- | --- | --- | --- | --- | --- | --- | --- |
|  |  | **Day 4** | **Day 7** | **BAL day** | **BAL time** |  |  | |  |
| 1 | 4 | 6.32 | 6.27 | 6.30 | 5.33 | 0.734 | 0.138 | |  |
| 2 | 2 | 4.26 | - | 2.79 | 3.56 | 2.002 | 0.562 | |  |
| 3 | 2 | 6.31 | 5.96 | 2.78 | 2.77 | 0.425 | 0.153 | |  |
| 4 | - | 6.28 | 6.84 | - | - | - | - | |  |
| 5 | - | 4.53 | 7.16 | - | - | - | - | |  |
| 6 | 24 | 2.49 | 2.57 | 8.26 | 6.98 | 3.302 | 0.473 | |  |
| 7 | 4 | 1.70 | 2.48 | 4.10 | 4.1 | 3.444 | 0.840 | |  |
| 8 | 24 | 2.51 | 2.24 | - | 5.64 | 2.321 | 0.411 | |  |
| 9 | 4 | 2.64 | 3.23 | 2.64 | 6.91 | 0.882 | 0.128 | |  |
| 10 | - | 2.23 | 3.07 | - | - | - | - | |  |
| 11 | 4 | 1.97 | 3.04 | - | 5.76 | 2.497 | 0.433 | |  |
| 12 | 2 | 2.60 | 2.52 | 2.21 | 3.56 | 0.481 | 0.135 | |  |
| 13 | 4 | 3.03 | 3.38 | 4.40 | 3.48 | 3.146 | 0.904 | |  |
